# Method-Specific Suicide Rates and Accessibility of Means: A Small-Area Analysis in Taipei City, Taiwan

**DOI:** 10.1027/0227-5910/a000793

**Published:** 2021-05-18

**Authors:** Chien-Yu Lin, Chia-Yueh Hsu, Ying-Yeh Chen, Shu-Sen Chang, David Gunnell

**Affiliations:** ^1^ Institute of Health Behaviors and Community Sciences, College of Public Health, National Taiwan University, Taipei, Taiwan; ^2^ Graduate School of Sport Sciences, Waseda University, Tokorozawa, Japan; ^3^ Department of Psychiatry, Wan Fang Hospital, Taipei Medical University, Taipei, Taiwan; ^4^ Department of Psychiatry, School of Medicine, College of Medicine, Taipei Medical University, Taipei, Taiwan; ^5^ Psychiatric Research Center, Wan Fang Hospital, Taipei Medical University, Taipei, Taiwan; ^6^ Taipei City Psychiatric Centre, Taipei City Hospital, Taipei, Taiwan; ^7^ Institute of Public Health and Department of Public Health, National Yang-Ming University, Taipei, Taiwan; ^8^ Centre for Academic Mental Health, Population Health Sciences, University of Bristol, UK; ^9^ National Institute of Health Research Biomedical Research Centre at the University Hospitals Bristol and Weston NHS Foundation Trust and the University of Bristol, UK

**Keywords:** suicide, suicide methods, spatial analysis, means accessibility

## Abstract

**Abstract.** *Background:* Few studies have investigated whether means accessibility is related to the spatial distribution of suicide. *Aims:* To examine the hypothesis that indicators of the accessibility to specific suicide methods were associated with method-specific suicide rates in Taipei City, Taiwan. *Method:* Smoothed standardized mortality ratios for method-specific suicide rates across 432 neighborhoods and their associations with means accessibility indicators were estimated using Bayesian hierarchical models. *Results:* The proportion of single-person households, indicating the ease of burning charcoal in the home, was associated with charcoal-burning suicide rates (adjusted rate ratio [aRR] = 1.13, 95% credible interval [CrI] = 1.03–1.25). The proportion of households living on the sixth floor or above, indicating easy access to high places, was associated with jumping suicide rates (aRR = 1.16, 95% CrI, 1.04–1.29). Neighborhoods’ adjacency to rivers, indicating easy access to water, showed no statistical evidence of an association with drowning suicide rates (aRR = 1.27, 95% CrI = 0.92–1.69). Hanging and overall suicide rates showed no associations with any of these three accessibility indicators. *Limitations:* This is an ecological study; associations between means accessibility and suicide cannot be directly inferred as causal. *Conclusion:* The findings have implications for identifying high-risk groups for charcoal-burning suicide (e.g., vulnerable individuals living alone) and preventing jumping suicides by increasing the safety of high buildings.

Preventing suicide is a global public health priority. Worldwide, an estimated 800,000 people die by suicide each year (World Health Organization, 2018). Previous time trend studies show that changes in the popularity and accessibility of means for suicide, such as coal or car exhaust gases (Thomas et al., 2011) or pesticides (Gunnell et al., 2007), may influence suicide rates. Person-based studies also indicate that easy access to lethal means such as firearms is related to individuals’ suicide risk (Barber & Miller, 2014). These studies have informed suicide prevention strategies involving the restriction of access to lethal means (Yip et al., 2012). However, fewer studies have examined spatial variations in suicide rates of various methods in relation to means accessibility. The identification of factors associated with such variations could inform local suicide prevention programs.

Previous studies from Taiwan and the United States showed that areas with higher levels of agricultural workers (i.e., an indicator of accessibility to pesticides) or household firearm ownership (i.e., an indicator of accessibility to firearms) had higher suicide rates by pesticide poisoning (Chang et al., 2012; Lin & Lu, 2006) or firearms (Opoliner et al., 2014), respectively. These studies were based on rather large geographic regions and did not focus on urban areas, although the majority of the world’s population now live in urban areas (United Nations, 2019) and past studies reported large variations in suicide rates within cities (Gotsens et al., 2013; Lin et al., 2019). However, factors associated with geographical variations in method-specific suicide rates in urban settings have rarely been studied. Marzuk et al. (1992) compared suicide incidence across five boroughs in New York City in 1984–1987 and found that the differences in method-specific suicide rates between boroughs might be largely due to differences in accessibility to lethal methods of suicide such as jumping from a height.

The use of some suicide methods such as charcoal burning, jumping, and drowning increased recently in some Asian cities. Suicides from carbon monoxide poisoning by burning barbecue charcoal in a sealed space increased markedly in Hong Kong and urban Taiwan from the late 1990s (Liu et al., 2007). A recent study showed that people who died by charcoal-burning suicide were more likely to live alone than those who killed themselves using other methods (Chang et al., 2019). The ease of implementing charcoal burning without discovery or interruption in the home could be a possible contributing factor to the increased risk of charcoal-burning suicide in individuals living alone. Therefore, the proportion of individuals living alone could be associated with area variations in charcoal-burning suicide, although, to date, this has not been investigated.

Jumping from high-rise buildings is the main method for suicide in Hong Kong and Singapore (Yip & Tan, 1998), where more than 80% of the residents live in high buildings. In Taiwan, there was an increase in suicide by jumping from the early 1990s onward (Lin & Lu, 2008), and a previous study showed a relationship between the proportion of households living in high-rise accommodation and jumping suicide rates across 23 cities/counties in Taiwan (Lin & Lu, 2006). However, it is unclear whether the proportion of individuals living at high levels is related to area variations in jumping suicide rates *within* cities.

Easy access to water may increase the risk of suicide by drowning; alternatively, being exposed to blue spaces, that is, visible waterbodies or surface waters including streams/rivers in cities (Britton et al., 2020), may reduce stress and negative feelings and in turn improve mental health and decrease suicide risk (Helbich et al., 2018). However, there are no previous studies examining whether proximity to rivers is specifically associated with increased use of drowning as a method of suicide. A previous study from Taiwan showed increased incidence of suicide by drowning in coastal areas and cities with harbors or located close to major rivers (Chang et al., 2011). A recent study from two coastal areas of Vietnam, where there were a high number of drowning deaths in young populations, showed an association between living in close proximity to a river and increased overall mortality rate (Kim et al., 2014). By contrast, another study from the Netherlands showed no association between proximity to blue spaces and overall suicide rates (Helbich et al., 2018).

We investigated the associations between indicators of means accessibility and method-specific suicide rates in Taipei City, Taiwan (population: 2.7 million). Specifically, we aimed to examine the hypothesis that the three indicators of the opportunity of implementing or accessing particular methods of suicide, namely (i) the proportion of single-person households, (ii) the proportion of households living on the sixth floor or above, and (iii) a neighborhood’s adjacency to rivers, were specifically associated with area rates of suicide by charcoal burning, jumping, and drowning, respectively, but not associated with rates of suicide by hanging.

## Methods

### Suicide and Population Data

Suicide data were extracted from the cause-of-death mortality files among people aged 10 years and above in Taipei City during 2004–2010. Suicide deaths were identified using the International Classification of Diseases, Tenth Revision (ICD-10) codes X60-X84. Deaths of undetermined intent (Y10-Y34), injury due to suffocation (W75, W76, W83, and W84), and injury due to pesticide poisoning (X48) were also identified as previous studies from Taiwan indicated that many deaths in these categories were likely to be misclassified suicides (Chang et al., 2010). Certified and possible suicides combined are referred to as “suicides” throughout the paper.

We identified suicides using different suicide methods based on the following ICD-10 codes: X67 (charcoal burning), X70 (hanging), X71 (drowning), and X80 (jumping). Corresponding codes for undetermined death (Y17 for charcoal burning, Y20 for hanging, Y21 for drowning, and Y30 for jumping) and suffocation injury (W75, W76, W83, and W84 for hanging) were also used. Suicide and undetermined death of all other methods and injury of pesticide poisoning were classified as the “other methods” group. Each suicide was assigned to one of the “neighborhoods” (*n* = 432), an administrative geographic unit, based on the residential address recorded in the mortality data files. Due to the small numbers of suicides using some methods, data were aggregated over the entire 7-year study period. Nevertheless, there were still some neighborhoods with no suicides by specific methods (e.g., 58.1% of neighborhoods had no drowning suicides; Table A1 in the Appendix).

The population data were from the household registration data files. In Taiwan, there is a household registration system that provides monthly population statistics at the neighborhood level. The median neighborhood population aged 10 years and above was 5,050 (*M* = 5,500; *SD* = 2,400; range 840–31,300) in Taipei City over the study period.

The study was approved by the National Taiwan University Hospital Research Ethics Committee (201512030RINC).

### Data on Area-Level Characteristics

Data for median household income and the proportion of divorced/separated adults were extracted from Income Tax Statistics (2004–2010) and the 2000 census, respectively. We used these two variables to capture the neighborhoods’ levels of socioeconomic position and social fragmentation, respectively, which showed a strong and independent association with neighborhoods’ overall suicide rates in previous studies (Lin et al., 2019).

Three proxy indicators were used to measure the opportunity or accessibility of implementing specific suicide methods:

1. The neighborhood’s proportion of single-person households was used to measure the ease of using the charcoal burning method.
2. The neighborhood’s proportion of households living on the sixth floor or above was used to measure the accessibility to high places used for jumping (Lin & Lu, 2006).
3. A binary variable identifying whether or not a river flowed through the neighborhood or along its boundary was used to measure the accessibility to the drowning method. Among 432 Taipei City’s neighborhoods, 96 (22.2%) were defined as adjacent to rivers. We conducted a sensitivity analysis using a continuous variable of the direct distance from the center point (centroid) of each neighborhood to the nearest river.

Data for the proportions of single-person households and households living on the sixth floor or above were obtained from the 2000 census. The geographic data for neighborhood boundaries and rivers were obtained from the open data website of the Taipei City government (https://data.gov.tw/). The river accessibility variables, binary or continuous, were created using the join function in ArcGIS version 10.5.

### Statistical Analyses

Age-standardized (in 5-year age-bands) mortality ratios (SMRs) for suicide using different methods among people aged 10 years and above were calculated for each neighborhood. We used Bayesian hierarchical models to estimate the ‘smoothed’ SMRs and examine the associations between means accessibility indicators and method-specific suicide rates. The Bayesian hierarchical model is a Poisson regression model with two random effects accounting for the heterogeneity of suicide rates across Taipei City (i.e., global [unstructured] variability across the whole study region) as well as between the neighboring areas (i.e., local [structured] variability across neighborhoods sharing a common boundary) (Besag et al., 1991; Congdon, 1997). The dependent variable was the number of suicides in each neighborhood, and the expected number of suicides was included as the offset in the model. The standard errors of unstructured and structured variability were specified using a uniform distribution (0, 5) in the main analysis (Gelman, 2006) and using a gamma distribution (0.01, 0.01) for the inverse of the variance as the alternative prior in the sensitivity analysis (Mollie, 2001). The standardized values (i.e., *z* scores) of studied variables, or their log-transformed values if the raw values were skewed, were used in the regression analyses. Rate ratios (RRs) and their 95% credible intervals (CrIs) were estimated. Adjusted RRs (aRRs) for means accessibility indicators were estimated in models adjusting for the two socioeconomic characteristics (i.e., median household income and the proportion of divorced/separated adults). Bayesian hierarchical models were estimated using the Markov-chain Monte Carlo method (Gilks et al., 1996) in WinBUGS version 1.4 (Speigelhalter et al., 2003). To ensure the stability of Bayesian posterior processes, we examined both the graphical histories of the simulated rate ratios and the Gelman–Rubin convergence statistics (Gelman, 2006). Details of model estimations and the results of sensitivity analyses are provided in the Appendix.

## Results

There were 2,994 suicides among people aged 10 years and above (65.3% males) in Taipei City (2004–2010) and the annual rate of suicide was 18.1 per 100,000 population aged 10 years and above. Deaths by hanging (*n* = 892; 29.8%), charcoal burning (*n* = 859; 28.7%), jumping (*n* = 566; 18.9%), and drowning (*n* = 275; 9.2%) accounted for 86.6% of all suicides (Table 1). Table A1 in the Appendix shows the distribution of neighborhoods’ suicide numbers by method. After excluding the 10% extreme values, there was still a 1.94-fold difference in the SMRs for suicide across the 432 neighborhoods – the mid-90% ratio (i.e., the value of the 95th centile of the SMRs divided by the value 5th centile) of suicide SMRs was 1.94 (Table 1). Such a geographic variation was largest for drowning (mid-90% ratio = 3.77), followed by charcoal burning (2.65), hanging (1.53), other methods (2.01), and jumping (1.20).

**Table 1:** Summary statistics of the distribution of smoothed SMRs for suicide^a^ of different methods across 432 neighborhoods in Taipei City, 2004–2010.

	*n*	%	*M*	*SD*	5%	Median	95%	90% ratio^b^
Overall	2,994	100	1.01	0.21	0.74	0.97	1.43	1.94
Hanging	892	29.8	1.01	0.14	0.83	0.98	1.27	1.53
Charcoal burning	859	28.7	1.00	0.31	0.60	0.94	1.58	2.65
Jumping	566	18.9	1.00	0.05	0.93	0.99	1.11	1.20
Drowning	275	9.2	1.02	0.47	0.55	0.89	2.07	3.77
Other	402	13.4	1.01	0.23	0.73	0.95	1.47	2.01
*Note.* SMR = standardized mortality ratio. ^a^Including deaths certified either as suicide, undetermined death, or injury due to suffocation or pesticide poisoning. ^b^Differences over the 90% mid-range, that is, the value at 95% divided by the value at 5%.								

**Figure 1:**
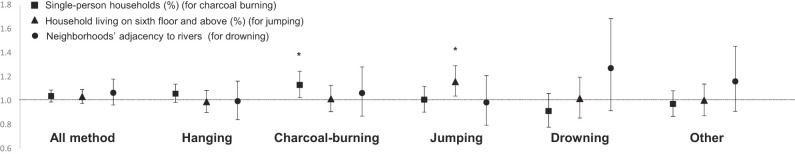
Rate ratios (RRs) and 95% credible intervals (CrIs) of method-specific suicide rates associated with one *SD* increase in the levels of each of the three means accessibility indicators across 432 neighborhoods in Taipei City, 2004–2010. *95% CrIs of RRs that do not include one.

Table 2 shows the associations of method-specific suicide rates with area socioeconomic characteristics and means accessibility indicators. The proportion of divorced/separated adults was positively associated with overall and method-specific suicide rates (except hanging and other methods), while median household income was negatively associated with overall and method-specific suicide rates (except jumping). After adjusting for these two socioeconomic characteristics, charcoal-burning suicide rates were specifically associated with single-person households (aRR = 1.13, 95% CrI = 1.03–1.25), while jumping suicide rates were specifically associated with households living on the sixth floor or above (aRR = 1.16, 95% CrI = 1.04–1.29). By contrast, no associations were found between drowning suicide rates and neighborhoods’ adjacency to rivers (aRR = 1.27, 95% CrI = 0.92–1.69; Figure 1). The sensitivity analysis using distance from neighborhood centroid to the nearest river also showed no association (aRR = 0.93, 95% CrI = 0.78–1.08; Table A2 in the Appendix). Neighborhoods’ overall suicide rates and suicide rates by hanging and other methods showed no associations with any of the three accessibility indicators. The sensitivity analysis using alternative priors showed similar results (Table A2 in the Appendix).

**Table 2:** RRs and 95% CrIs^a^ of method-specific suicide rates^b^ associated with one *SD* increase in the levels of each of the three means accessibility indicators across 432 neighborhoods in Taipei City, 2004–2010.

	All		Hanging		Charcoal burning		Jumping		Drowning		Other	
	RR	95% CrI	RR	95% CrI	RR	95% CrI	RR	95% CrI	RR	95% CrI	RR	95% CrI
**Unadjusted analysis**												
Socioeconomic characteristics												
Divorced/separated adults (%)^c^	**1.10**	**1.05, 1.16**	1.03	0.96, 1.11	**1.21**	**1.11, 1.31**	**1.14**	**1.04, 1.25**	**1.16**	**1.00, 1.34**	1.03	0.92, 1.15
Median household income^c^	**0.81**	**0.77, 0.85**	**0.80**	**0.74, 0.86**	**0.77**	**0.70, 0.84**	1.00	0.92, 1.10	**0.62**	**0.54, 0.71**	**0.82**	**0.74, 0.91**
Means accessibility indicators (for specific suicide methods)												
Single-person households (%^c^; for charcoal burning)	**1.10**	**1.05, 1.17**	**1.11**	**1.02, 1.21**	**1.24**	**1.12, 1.36**	1.05	0.96, 1.15	1.01	0.85, 1.19	1.00	0.89, 1.12
Household living on sixth floor or above (%; for jumping)	0.96	0.91, 1.02	**0.88**	**0.81, 0.95**	0.96	0.87, 1.05	**1.15**	**1.05, 1.25**	0.86	0.73, 1.01	**0.90**	**0.80, 1.00**
Neighborhoods with river (for drowning)	**1.17**	**1.04, 1.32**	1.15	0.96, 1.35	1.17	0.95, 1.43	0.96	0.78, 1.18	**1.59**	**1.11, 2.18**	**1.31**	**1.03, 1.63**
**Adjusted analysis** ^d^												
Means accessibility indicators (for specific suicide methods)												
Single-person households (%^c^; for charcoal burning)	1.04	0.99, 1.09	1.06	0.98, 1.14	**1.13**	**1.03, 1.25**	1.01	0.90, 1.12	0.91	0.78, 1.06	0.97	0.87, 1.08
Household living on sixth floor or above (%; for jumping)	1.03	0.98, 1.09	0.99	0.90, 1.09	1.02	0.91, 1.13	**1.16**	**1.04, 1.29**	1.02	0.86, 1.20	1.00	0.87, 1.14
Neighborhoods with river (for drowning)	1.07	0.96, 1.18	1.00	0.84, 1.16	1.06	0.87, 1.28	0.99	0.80, 1.21	1.27	0.92, 1.69	1.16	0.91, 1.45
*Note.* CrI = credible interval; RR = rate ratio. ^a^95% CrIs of RRs that do not include one are highlighted in bold. ^b^Including deaths certified as suicide, undetermined death, or injury due to suffocation or pesticide poisoning. ^c^These variables were first log-transformed because of their skewed distributions. ^d^Adjusted for the proportion of divorced/separated adults and median household income.												

## Discussion

We found evidence that access to means (or opportunity to use means) was associated with method-specific suicide rates across neighborhoods for charcoal burning (accessibility indicated by the proportion of single-person households) and jumping (accessibility indicated by the proportion of households living on the sixth floor or above). By contrast, there was no evidence for an association between neighborhoods’ proximity to rivers and drowning suicide rates. Furthermore, there were no associations of neighborhoods’ suicide rates of hanging (as a control method) with any of the means accessibility indicators. Finally, the three access-to-means indicators were not associated with a neighborhood’s overall suicide rate, indicating that prevention strategies focusing on these methods may not have an impact on overall suicide rates.

### Comparison With Previous Studies

Our data showed an association between the proportion of single-person households and neighborhood charcoal-burning suicide rates. In keeping with this, a recent study of 11,854 suicides from Hong Kong showed that, when compared with people who died by suicide using other methods, those who burnt charcoal were approximately three times more likely to live alone even after adjusting for age and sex (Chang et al., 2019). Of note, in previous ecological studies of suicide, an area’s proportion of single-person households was also commonly used as an indicator of *social fragmentation*, namely “anomie” or the breakdown of social integration, which was theorized by Durkheim as the connectedness between individuals and society that may decrease population suicide rates (Congdon, 2004). However, in our analysis, this indicator was only associated with charcoal-burning suicide rates, but not the rates of overall suicide or suicide using other non-charcoal-burning methods, suggesting that this indicator may specifically capture the neighborhood level of ease of using the charcoal-burning method rather than social fragmentation in Taipei City. This finding suggests that vulnerable individuals who live alone could be a high-risk group for charcoal-burning suicide, particularly in countries where the method is commonly used (Chang et al., 2014). In Cheung Chau, an island of Hong Kong, the owners and managers of holiday flats in the island refused to rent rooms to individuals who traveled alone and/or appeared depressed to prevent them from attempting suicide by burning charcoal in the rented unit (Wong et al., 2009). A decrease in annual visitor suicides was reported after implementing this intervention (Wong et al., 2009). Strategies aimed at identifying high-risk individuals and reducing the likelihood that they are alone at time of risk could be potentially effective approaches to preventing charcoal-burning suicide.

In keeping with previous studies (Lin & Lu, 2006; Panczak et al., 2013), our data showed a positive association between the proportion of households living on the sixth floor or above and jumping suicide rates. A recent systematic review showed that installing barriers at popular sites for jumping was followed by a decrease in suicide by this method (Pirkis et al., 2015; Zalsman et al., 2016); however, it may be challenging to restrict access to high places in some urban settings where a large proportion of the population live in high-rise buildings. A population-based approach may be needed to enhance the safety of all residential buildings by enacting and enforcing laws or regulations that require the installation of high fences or safety nets at the roofs or balconies of the buildings as well as window guards throughout the buildings. However, this may require considerations of the balance between liberty/household comfort and preventing suicide, which is a relatively rare event.

Our data showed no statistical evidence for an association of drowning suicide rates with neighborhoods’ adjacency to rivers, although the direction of association was consistent with increased risk for those living nearby. However, drowning suicides could occur at sites other than rivers. A recent report from Taipei City showed that, although 78% of drowning suicides were found to occur in rivers, other common sites for drowning suicide included a wide range of locations such as ponds, harbors, and reservoirs (Chang, 2017). Furthermore, the physical proximity from each neighborhood to rivers might not be the most appropriate accessibility indicator in the city because of the ease to access rivers using public or private transport. Future research could examine the locations where drowning suicides commonly occurred to identify high-risk spots and develop more accurate indicators of accessibility to potential sites for drowning suicide. Potential interventions might include enhancing the safety of these sites and increasing the chance of rescue by installing more lifebelts and emergency services.

Our data showed that none of the method-specific accessibility indicators investigated was associated with overall suicide rates. The result suggests that targeting on these suicide methods may have limited impact on overall suicide rates in Taipei City. By contrast, previous studies investigating accessibility indicators of firearms (Opoliner et al., 2014) and pesticide poisoning (Chang et al., 2012) showed an association with both method-specific and overall suicide rates. The difference in findings could be due to the difference in the share of method-specific suicide investigated among overall suicides. In previous studies of suicide by firearms (Opoliner et al., 2014) and pesticide poisoning (Chang et al., 2012), the two methods accounted for a large proportion of suicides in the whole or specific study regions – approximately half of suicides in the United States (Opoliner et al., 2014) and East and Central Taiwan (Chang et al., 2012), respectively. By contrast, the proportion of suicides by each of the methods investigated in this study was lower than 30% (ranging from 9.2% for drowning to 28.7% for charcoal burning).

Some previous studies have shown higher suicide rates in rural areas than urban areas in countries such as Taiwan (Chang et al., 2011) and Germany (Helbich et al., 2017); however, in Taiwan, most suicides occurred in urban areas as a much larger proportion of population live in urban than rural areas (Chang et al., 2011), therefore highlighting the importance of suicide prevention in cities. Our findings provide new insights into means accessibility and suicide in a rapidly growing Asian city; these would have important implications for other fast-growing cities around the world, many of which are similarly characterized by a marked increase in residents living in high-rise buildings and/or living alone, and this may increase the risk of suicide by jumping or other methods that may be challenging to implement in shared accommodation, such as charcoal burning.

## Strengths and Limitations

To the best of our knowledge, this is among the first detailed analyses of associations between the indicators of ease of using or accessing suicide means with small-area variations in method-specific suicides rates in cities. There are several limitations to this study. First, this is an ecological study, and its findings may not apply at the individual level, and the associations between means accessibility and suicide cannot be directly inferred as causal. The neighborhoods analyzed in the study were administrative units, and the results may differ if data are aggregated at different geographic units (i.e., the modifiable areal unit problem; Tate & Atkinson, 2001). Second, we used single-person households as an indicator for the ease of using the charcoal-burning method; however, the opportunity of implementing the method in single-person households may be influenced by other characteristics such as the size of the dwelling (Goldstein, 2008). Furthermore, the likelihood of suicide by drowning may be influenced by not only the proximity to rivers but also the size (i.e., width and depth) of rivers; however, the size of rivers was not considered in the analysis as relevant data were unavailable. Third, we used the residential address before death when calculating suicide rates at the neighborhood level, while some of the suicides may not have occurred at the residential address. For example, some charcoal-burning suicides occurred in cars or hotel rooms (Chang, 2017); some jumping suicides occurred at nonresidential buildings such as shopping malls (Chen et al., 2009); and most drowning suicides occurred outside home (Chang, 2017). However, in Taipei City, it was reported that the majority of charcoal-burning suicides (71%) occurred at home (Chang, 2017), and most jumping suicides (67%) occurred at residential buildings (Chen et al., 2009). Furthermore, most drowning suicides (78%) involved drowning in rivers in Taipei City (Chang, 2017), and we used the adjacency to rivers as the accessibility indicator for drowning suicide in the study.

## Conclusion

In conclusion, our small-area analysis showed an association of the ease of using or accessing specific lethal means with geographic variations in method-specific suicide rates. Vulnerable individuals living alone could be considered as a high-risk group for charcoal-burning suicide in countries where the method is commonly used for suicide. A systematic, population-based approach may be needed to enhance the safety of high-rise residential buildings to meaningfully reduce jumping suicide rates. Potential approaches include enforcing laws that mandate the installation of high fences, safety net, and window guards. Future research needs to better understand the spatial distributions and physical characteristics of potential sites for suicide using methods such as charcoal burning, jumping, and drowning to inform prevention strategies.

## Notes

**Publication Ethics:** The study was approved by the National Taiwan University Hospital Research Ethics Committee (201512030RINC).

## Appendix

### 1. Bayesian Regression Model Description

We used noninformative prior distributions for the specifications of unstructured and structured variability in the Bayesian models. The standard errors of unstructured and structured variability were specified using a uniform distribution (0, 5) in the main analysis (Gelman, 2006), and using a gamma distribution (0.01, 0.01) for the inverse of the variance as an alternative prior in the sensitivity analysis (Mollie, 2001). All models were run for an initial ‘burn-in’ period of 10,000 iterations, and these ‘burn-in’ samples were discarded; the statistics of the posterior estimates were derived based on a total sample of 22,500 iterations pooled from three chains with different initials (i.e., each chain was run for a further 7,500 iterations after the first 10,000 iterations).

### 2. Descriptive Statistics of the Suicide Data

Table A1 shows the summary statistics for the distribution of the number of suicides by method across neighborhoods during the study period.

**Table A1:** Summary statistics of the distribution of the number of suicides^a^ for people aged 10+ across 432 neighborhoods in Taipei City, 2004–2010.

	Sum	Mean number per area	*SD*	Neighborhoods with no suicides (%)	Min	5%	25%	Median	75%	95%	Max
Males and females											
All methods combined	2,994	6.93	4.03	4 (0.9)	0	2	4	6	9	14	31
Hanging	892	2.06	1.59	56 (13.0)	0	0	1	2	3	5	9
Charcoal-burning	859	1.99	1.81	94 (21.8)	0	0	1	2	3	5	12
Jumping	566	1.31	1.27	128 (29.6)	0	0	0	1	2	4	6
Drowning	275	0.64	0.95	251 (58.1)	0	0	0	0	1	2	6
Other	402	0.93	1.01	177 (41.0)	0	0	0	1	1	3	5
^a^Including deaths certified as suicide, undetermined death, or injury due to suffocation or pesticide poisoning.											

### 3. Results of Sensitivity Analyses

Table A2 shows the results of sensitivity analysis using alternative priors in the Bayesian hierarchical models (part A) and using a distance-based measure of proximity to rivers (part B).

**Table A2:** aRRs^a^ and 95% CrIs^b^ of method-specific suicide rates^c^ associated with one *SD* increase in levels of each of the three means accessibility indicators across 432 neighborhoods in Taipei City, 2004–2010: sensitivity analyses (A) using gamma distribution (0.01, 0.01) priors for the inverse of the variance of the unstructured and structured variability in the Bayesian hierarchical model and (B) using the distance from neighborhood centroids to the nearest river as the accessibility indicator for drowning.

	All		Hanging		Charcoal burning		Jumping		Drowning		Other	
	aRR	95% CrI	aRR	95% CrI	aRR	95% CrI	aRR	95% CrI	aRR	95% CrI	aRR	95% CrI
**(A)**												
Means accessibility indicators (for specific suicide methods)												
Single-person households (%^d^; for charcoal burning)	1.04	0.99, 1.10	1.06	0.98, 1.14	**1.12**	**1.01, 1.24**	1.01	0.92, 1.12	0.93	0.80, 1.06	0.98	0.87, 1.09
Household living on sixth floor or above (%; for jumping)	1.03	0.98, 1.09	0.99	0.90, 1.08	1.01	0.91, 1.11	**1.16**	**1.04, 1.29**	1.02	0.85, 1.20	1.01	0.88, 1.14
Neighborhoods with river (for drowning)	1.08	0.97, 1.20	1.00	0.84, 1.19	1.07	0.88, 1.28	0.99	0.79, 1.22	1.26	0.93, 1.66	1.16	0.91, 1.46
**(B)**												
Means accessibility indicators (for specific suicide methods)												
Single-person households (%^d^; for charcoal burning)	1.04	0.99, 1.09	1.07	0.99, 1.16	**1.14**	**1.03, 1.26**	0.96	0.87, 1.06	0.92	0.78, 1.08	0.98	0.87, 1.10
Household living on sixth floor or above (%; for jumping)	1.02	0.96, 1.08	0.96	0.87, 1.06	0.97	0.87, 1.08	**1.18**	**1.05, 1.31**	1.07	0.88, 1.27	1.03	0.89, 1.18
Distance from neighborhood centroids to the nearest river (for drowning)	1.01	0.96, 1.06	1.07	0.99, 1.16	0.95	0.86, 1.06	1.07	0.96, 1.17	0.93	0.78, 1.08	0.95	0.84, 1.06
*Note.* aRR = adjusted rate ratio; CrI = credible interval; RR = rate ratio. ^a^Adjusted for the proportion of divorced/separated adults and median household income. ^b^95% CrIs of RRs that do not include one are highlighted in bold. ^c^Including deaths certified either as suicide, undetermined death, or injury due to suffocation or pesticide poisoning. ^d^These variables were first log-transformed because of their skewed distributions.												

## References

[c1] Barber, C. W., & Miller, M. J. (2014). Reducing a suicidal person's access to lethal means of suicide: A research agenda. *American Journal of Preventive Medicine*, *47*(3 suppl 2), S264–S272. 10.1016/j.amepre.2014.05.028 25145749

[c2] Besag, J., York, J., & Mollié, A. (1991). Bayesian image restoration, with two applications in spatial statistics. *Annals of the Institute of Statistical Mathematics*, *43*(1), 1–20. 10.1007/BF00116466

[c3] Britton, E., Kindermann, G., Domegan, C., & Carlin, C. (2020). Blue care: A systematic review of blue space interventions for health and wellbeing. *Health Promotion International*, *35*(1), 50–69. 10.1093/heapro/day103 30561661 PMC7245048

[c4] Chang, S. S. (2017). *Report of suicide prevention strategies in Taipei: An analysis of suicide in Taipei City 2012–2016*. Department of Health, Taipei City Government.

[c5] Chang, S.-S., Chen, Y.-Y., Yip, P. S. F., Lee, W. J., Hagihara, A., & Gunnell, D. (2014). Regional changes in charcoal-burning suicide rates in East/Southeast Asia from 1995 to 2011: A time trend analysis. *PLoS Medicine*, *11*(4), e1001622. 10.1371/journal.pmed.1001622 24691071 PMC3972087

[c7] Chang, S. S., Lu, T. H., Sterne, J. A., Eddleston, M., Lin, J. J., & Gunnell, D. (2012). The impact of pesticide suicide on the geographic distribution of suicide in Taiwan: A spatial analysis. *BMC Public Health*, *12*(1), 260. 10.1186/1471-2458-12-260 22471759 PMC3351735

[c8] Chang, S.-S., Sterne, J. A. C., Lu, T.-H., & Gunnell, D. (2010). ‘Hidden’ suicides amongst deaths certified as undetermined intent, accident by pesticide poisoning and accident by suffocation in Taiwan. *Social Psychiatry and Psychiatric Epidemiology*, *45*(2), 143–152. 10.1007/s00127-009-0049-x 19363577

[c9] Chang, S.-S., Sterne, J. A. C., Wheeler, B. W., Lu, T.-H., Lin, J.-J., & Gunnell, D. (2011). Geography of suicide in Taiwan: Spatial patterning and socioeconomic correlates. *Health & Place*, *17*(2), 641–650. 10.1016/j.healthplace.2011.01.003 21292534

[c6] Chang, Y.-H., Hsu, C.-Y., Cheng, Q., Chang, S.-S., & Yip, P. (2019). The evolution of the characteristics of charcoal-burning suicide in Hong Kong, 2002–2013. *Journal of Affective Disorders*, *257*, 390–395. 10.1016/j.jad.2019.07.041 31306989

[c10] Chen, Y.-Y., Gunnell, D., & Lu, T.-H. (2009). Descriptive epidemiological study of sites of suicide jumps in Taipei, Taiwan. *Injury Prevention*, *15*(1), 41–44. 10.1136/ip.2008.019794 19190275

[c11] Congdon, P. (1997). Bayesian models for spatial incidence: A case study of suicide using the BUGS program. *Health & Place*, *3*(4), 229–247. 10.1016/S1353-8292(97)00017-8

[c12] Congdon, P. (2004). Commentary: Contextual effects: Index construction and technique. *International Journal of Epidemiology*, *33*(4), 741–742. 10.1093/ije/dyh173 15155697

[c13] Gelman, A. (2006). Prior distributions for variance parameters in hierarchical models (comment on article by Browne and Draper). *Bayesian Analysis*, *1*(3), 515–534. 10.1214/06-Ba117a

[c14] Gilks, W. R., Richardson, S., & Spiegelhalter, D. J. (1996). *Markov chain Monte Carlo in practice*. Chapman & Hall.

[c15] Goldstein, M. (2008). Carbon monoxide poisoning. *Journal of Emergency Nursing*, *34*(6), 538–542. 10.1016/j.jen.2007.11.014 19022078

[c16] Gotsens, M., Marí-Dell'Olmo, M., Pérez, K., Palència, L., Martinez-Beneito, M.-A., Rodríguez-Sanz, M., Burström, B., Costa, G., Deboosere, P., Domínguez-Berjón, F., Dzúrová, D., Gandarillas, A., Hoffmann, R., Kovacs, K., Marinacci, C., Martikainen, P., Pikhart, H., Rosicova, K., …, Borrell C. (2013). Socioeconomic inequalities in injury mortality in small areas of 15 European cities. *Health & Place*, *24*, 165–172. 10.1016/j.healthplace.2013.09.003 24112963

[c17] Gunnell, D., Fernando, R., Hewagama, M., Priyangika, W. D. D., Konradsen, F., & Eddleston, M. (2007). The impact of pesticide regulations on suicide in Sri Lanka. *International Journal of Epidemiology*, *36*(6), 1235–1242. 10.1093/ije/dym164 17726039 PMC3154644

[c18] Helbich, M., Bluml, V., de Jong, T., Plener, P. L., Kwan, M. P., & Kapusta, N. D. (2017). Urban–rural inequalities in suicide mortality: A comparison of urbanicity indicators. *International Journal of Health Geography*, *16*(1), 39. 10.1186/s12942-017-0112-x PMC5663034 29084555

[c19] Helbich, M., de Beurs, D., Kwan, M.-P., O'Connor, R. C., & Groenewegen, P. P. (2018). Natural environments and suicide mortality in The Netherlands: A cross-sectional, ecological study. *The Lancet Planetary Health*, *2*(3), e134–e139. 10.1016/S2542-5196(18)30033-0 29546252 PMC5846805

[c20] Kim, D. R., Ali, M., Thiem, V. D., & Wierzba, T. F. (2014). Socio-ecological risk factors for prime-age adult death in two coastal areas of Vietnam. *PLoS One*, *9*(2), e89780. 10.1371/journal.pone.0089780 24587031 PMC3935940

[c21] Lin, C.-Y., Hsu, C.-Y., Gunnell, D., Chen, Y.-Y., & Chang, S.-S. (2019). Spatial patterning, correlates, and inequality in suicide across 432 neighborhoods in Taipei City, Taiwan. *Social Science & Medicine*, *222*, 20–34. 10.1016/j.socscimed.2018.12.011 30580123

[c22] Lin, J.-J., & Lu, T.-H. (2006). Association between the accessibility to lethal methods and method-specific suicide rates: An ecological study in Taiwan. *Journal of Clinical Psychiatry*, *67*(7), 1074–1079. 10.4088/jcp.v67n0709 16889450

[c23] Lin, J. J., & Lu, T. H. (2008). Suicide mortality trends by sex, age and method in Taiwan, 1971–2005. *BMC Public Health*, *8*(1), 6. 10.1186/1471-2458-8-6 18179723 PMC2259336

[c24] Liu, K. Y., Beautrais, A., Caine, E., Chan, K., Chao, A., Conwell, Y., LawLee, C.D., Lee, D., Li, P., & Yip, P. (2007). Charcoal burning suicides in Hong Kong and urban Taiwan: An illustration of the impact of a novel suicide method on overall regional rates. *Journal of Epidemiology & Community Health*, *61*(3), 248–253. 10.1136/jech.2006.048553 17325404 PMC2652925

[c25] Marzuk, P. M., Leon, A. C., Tardiff, K., Morgan, E. B., Stajic, M., & Mann, J. J. (1992). The effect of access to lethal methods of injury on suicide rates. *Archives of General Psychiatry*, *49*(6), 451–458. 10.1001/archpsyc.1992.01820060031005 1599369

[c26] Mollie, A. (2001). Bayesian mapping of Hodgkin's disease in France. In P. Elliot, J. C. Wakefield, N. G. Best, & D. J. Briggs (Eds.), Spatial epidemiology: Methods and applications (pp. 267–285). Oxford University Press.

[c27] Opoliner, A., Azrael, D., Barber, C., Fitzmaurice, G., & Miller, M. (2014). Explaining geographic patterns of suicide in the US: The role of firearms and antidepressants. *Injury Epidemiology*, *1*(1), 6. 10.1186/2197-1714-1-6 27747669 PMC5005708

[c28] Panczak, R., Galobardes, B., Spoerri, A., Zwahlen, M., & Egger, M. (2013). High life in the sky? Mortality by floor of residence in Switzerland. *European Journal of Epidemiology*, *28*(6), 453–462. 10.1007/s10654-013-9809-8 23661152 PMC3696174

[c38] Pirkis, J., Too, L. S., Spittal, M. J., Krysinska, K., Robinson, J., & Cheung, Y. T. (2015). Interventions to reduce suicides at suicide hotspots: A systematic review and meta-analysis. *Lancet Psychiatry*, *2*(11), 994–1001. 10.1016/S2215-0366(15)00266-7 26409438

[c29] Speigelhalter, D., Thomas, A., Best, N., & Lunn, D. (2003). *WinBUGS User Manual, Version 1.4*. https://www.mrc-bsu.cam.ac.uk/wp-content/uploads/manual14.pdf

[c30] Tate, N., & Atkinson, P. M. (2001). *Modelling scale in geographical information science*. John Wiley & Sons.

[c31] Thomas, K., Chang, S. S., & Gunnell, D. (2011). Suicide epidemics: The impact of newly emerging methods on overall suicide rates – A time trends study. *BMC Public Health*, *11*(1), 314. 10.1186/1471-2458-11-314 21569569 PMC3112128

[c32] United Nations, Department of Economic and Social Affairs, Population Division. (2019). World urbanization prospects 2018: Highlights. https://population.un.org/wup/Publications/Files/WUP2018-Highlights.pdf

[c33] Wong, P. W. C., Liu, P. M. Y., Chan, W. S. C., Law, Y. W., Law, S. C. K., Fu, K.-W., Li, H. S. H., Tso, M. K., Beautrais, A. L., & Yip, P. S. F. (2009). An integrative suicide prevention program for visitor charcoal burning suicide and suicide pact. *Suicide and Life-Threatening Behavior*, *39*(1), 82–90. 10.1521/suli.2009.39.1.82 19298153

[c34] World Health Organization. (2018). *Suicide fact sheets*. https://www.who.int/mental_health/prevention/suicide/estimates/en/

[c35] Yip, P. S., Caine, E., Yousuf, S., Chang, S.-S., Wu, K. C.-C., & Chen, Y.-Y. (2012). Means restriction for suicide prevention. *The Lancet*, *379*(9834), 2393–2399. 10.1016/S0140-6736(12)60521-2 PMC6191653 22726520

[c36] Yip, P. S. F., & Tan, R. C. E. (1998). Suicides in Hong-Kong and Singapore: A tale of two cities. *International Journal of Social Psychiatry*, *44*(4), 267–279. 10.1177/002076409804400403 10459510

[c37] Zalsman, G., Hawton, K., Wasserman, D., van Heeringen, K., Arensman, E., Sarchiapone, M., Carli, V., Höschl, C., Barzilay, R., Balazs, J., Purebl, G., Kahn, J. P., Sáiz, P. A., Lipsicas, C. B., Bobes, J., Cozman, D., Hegerl, U., & Zohar, J. (2016). Suicide prevention strategies revisited: 10-year systematic review. *The Lancet Psychiatry*, *3*(7), 646–659. 10.1016/S2215-0366(16)30030-X 27289303

